# Formulation Development and Evaluation of Hybrid Nanocarrier for Cancer Therapy: Taguchi Orthogonal Array Based Design

**DOI:** 10.1155/2013/712678

**Published:** 2013-09-11

**Authors:** Rakesh K. Tekade, Mahavir B. Chougule

**Affiliations:** Department of Pharmaceutical Science, College of Pharmacy, University of Hawaii at Hilo, 34 Rainbow Drive, Suite 300 Hilo, HI 96720, USA

## Abstract

Taguchi orthogonal array design is a statistical approach that helps to overcome limitations associated with time consuming full factorial experimental design. In this study, the Taguchi orthogonal array design was applied to establish the optimum conditions for bovine serum albumin (BSA) nanocarrier (ANC) preparation. Taguchi method with L9 type of robust orthogonal array design was adopted to optimize the experimental conditions. Three key dependent factors namely, BSA concentration (% w/v), volume of BSA solution to total ethanol ratio (v : v), and concentration of diluted ethanolic aqueous solution (% v/v), were studied at three levels 3%, 4%, and 5% w/v; 1 : 0.75, 1 : 0.90, and 1 : 1.05 v/v; 40%, 70%, and 100% v/v, respectively. The ethanolic aqueous solution was used to impart less harsh condition for desolvation and attain controlled nanoparticle formation. The interaction plot studies inferred the ethanolic aqueous solution concentration to be the most influential parameter that affects the particle size of nanoformulation. This method (BSA, 4% w/v; volume of BSA solution to total ethanol ratio, 1 : 0.90 v/v; concentration of diluted ethanolic solution, 70% v/v) was able to successfully develop Gemcitabine (G) loaded modified albumin nanocarrier (M-ANC-G) of size 25.07 ± 2.81 nm (*ζ* = −23.03 ± 1.015 mV) as against to 78.01 ± 4.99 nm (*ζ* = −24.88 ± 1.37 mV) using conventional method albumin nanocarrier (C-ANC-G). Hybrid nanocarriers were generated by chitosan layering (solvent gelation technique) of respective ANC to form C-HNC-G and M-HNC-G of sizes 125.29 ± 5.62 nm (*ζ* = 12.01 ± 0.51 mV) and 46.28 ± 2.21 nm (*ζ* = 15.05 ± 0.39 mV), respectively. Zeta potential, entrapment, *in vitro* release, and pH-based stability studies were investigated and influence of formulation parameters are discussed. Cell-line-based cytotoxicity assay (*A549* and *H460* cells) and cell internalization assay (*H460* cell line) were performed to assess the influence on the bioperformance of these nanoformulations.

## 1. Introduction

The term “nanotechnology” refers to the utilization of bio-material to produce novel nanoarchitecture with controlled shape, size, form, and properties for varying applications [1–4]. A wide array of polymeric- as well as lipid-based nanocarriers (NC) such as dendrimer, carbon nanotubes, and liposome have been explored but there are biodegradability, toxicity, and poor efficacy issues associated with them [3, 5, 6]. Liposomes have been realized as prospective drug carriers but its application has been greatly hampered due to associated size, encapsulation, and drug leakage, as well as stability issues [7, 8]. This mandates exploration of safe biomaterial for drug delivery, which can be successfully used in clinic.

Agreeing with Generally Regarded As Safe (GRAS) motif, an ideal nanocarrier must be nontoxic, and of biodegradable, nanosize range (preferably <100 nm), bears slight positive charge, must be stable under *in vivo* condition, retains drug during circulation, and releases the loaded drug selectively as well as rapidly under tumorous environment. In recent years, there has been a notable interest in the development of biodegradable nanocarriers as a drug delivery device [9, 10]. In this line, it is anticipated that biodegradable polymer-based nanocarriers can provide a way of sustained, controlled, and targeted drug delivery to improve the therapeutic effects and reduce the side effects of the formulated drugs [1, 11, 12].

Albumin is a biodegradable/biocompatible polymer approved by FDA for i.v. administration [15, 16]. Albumin-based nanocarrier (ANC) represents an attractive strategy, since a significant amount of bioactive can be incorporated into the nanocarriers because of the different drug binding sites present in it. Bae et al. formulated albumin nanocarriers of size ranging between 180 and 300 nm to deliver anticancer drug, doxorubicin [17]. Similarly Anhorn et al. have also been successful in developing the albumin nanoparticles of approximately 150 nm in size [18] and it was suggested therein to explore strategies to develop nanoparticles of small size range. Also, albumin nanocarriers are negatively charged [14, 18] and hence mandate their surface modification to achieve positive surface charge so as to maximize their interaction with negatively charged cancer cells [19, 20] Chitosan also represents another class of biodegradable, biocompatible, non-toxic biomaterial with ease for chemical modifications that suits more closely for making ligand anchoring (drug targeting) [15]. The use of albumin- and chitosan-based nanocarriers will help in the translational application of these nanocarriers. Hence, in this work, we have formulated the albumin- and chitosan-based hybrid nanocarrier formulation with ANC as core with surface topped positively charged chitosan layering for efficient cancer specific delivery of loaded drug.

The albumin-chitosan hybrid nanocarriers (HNC) approach to deliver a model anticancer drug Gemcitabine (GEM) reported in this paper is an innovative concept and has not been studied. Acetaminophen (APAP) is also engaged as a model drug for rigorous optimization owing to its appropriate nature as well as easy availability under laboratory settings. Chitosan-based hybrid nanoconstruct is prepared with an aim to enhance uptake by negatively charged cancerous cells via electrostatic interaction [4, 21]. The protonation of amine groups of chitosan in the acidic environment of tumors will further facilitate the release of loaded drug to achieve efficient delivery inside the cells and escapes of NC from endosomes to release loaded drug in cytosol [22]. Additionally, in order to attain rapid growth, tumor tissue undergoes neovascularization and angiogenesis for their amplified nutritional and oxygen requirements. These newly formed tumor vessels are usually abnormal and bear poorly aligned defective endothelial cells with wide fenestrations [23].

The enhanced permeability and retention (EPR) effect is the phenomenon by which certain architects (of size <100 nm) are inclined to accumulate in tumor tissue through these nanofenestrations [24, 25]. However, the reported methodologies for formulation of albumin nanocarriers by desolation technique showed particle size of  ≥100 nm [14, 17]. The chief notable problem in designing the albumin- and chitosan-based nanocarrier is to attain formulation of small-sized (<50 nm) albumin nanocarrier core. Success in experiments and/or technology mainly depends on a properly designed process and/or product. The properties of ANC are affected by various parameters such as initial BSA concentration and concentration of ethanol. To establish a clear-cut understanding as well as interrelationships between various dependent parameters is complex, and the analysis of this system for optimizing the factors using full factorial design is a time and labor consuming task.

The efficient analyses of this complex formulations process using statistical experimental design have been proposed by “Taguchi Orthogonal Array Design” in this paper [26, 27]. Taguchi orthogonal array methods are statistical methods developed by Dr. Genichi Taguchi to lessen the experimental attributes to improve the quality of manufactured products, and more recently the applicative arena has been expanded to medicines, chemistry, engineering, biotechnology, and pharmaceutics as well as marketing and advertising [28, 29]. This method provides a systematic approach for conducting experimentation to determine optimum experimental settings of selected design parameters. This approach pushes the product quality back to the design stage for the design of a product or process, which are insensitive to quality problems [14, 26]. Taguchi method can significantly diminish the number of experimental configurations by using orthogonal array design. This approach drastically reduces the cost incurred in research and development by concurrently assessing a large number of experimental parameters with most suited experimental sets [26].

The Taguchi design can effectively determine the effect of the factors on characteristic properties with end conclusion on inferring optimal formulation conditions [26, 28]. It uses the tables of orthogonal arrays and analysis of variance (ANOVA) which can estimate the effect of a factor on the characteristic properties. Conventional statistical experimental design can determine the optimum condition on the basis of the measured values of the characteristic properties, while Taguchi's experimental design (also known as a robust parameter design) does this on the basis of the variability of characteristic properties [26, 27, 30].

In this work, simple coacervation technique was employed for formulation of ANC as a colloidal drug delivery system and the key factors effecting formulation of ANC (namely, BSA concentration, ethanol addition, etc.) were considered in preparation of nanoformulation. Hybrid nanocarrier (HNC) was formulated by solvent gelation technique using chitosan polymer. It was anticipated that, because of versatile polyfunctional and adaptive architect, BSA-based ANC will load higher proportion of biofunctional bioactive (drug), while surface stabilized chitosan layering (in HNC) will help in attaining higher cellular internalization as well as delivery of significant amount of drug inside the cellular compartment.

The core objectives of the present study are (i) to optimize the formulation of BSA-based nanocarriers by the Taguchi design method which show a controllable particle diameter <50 nm and a narrow size distribution and (ii) to develop the hybrid nanocarriers by coating the preformed ANC by chitosan for efficient tumor cell internalization. In addition, this study also intends to establish a rational basis for the controlled production and application of protein-based nanocarriers as drug carrier systems.

**Figure 1:**
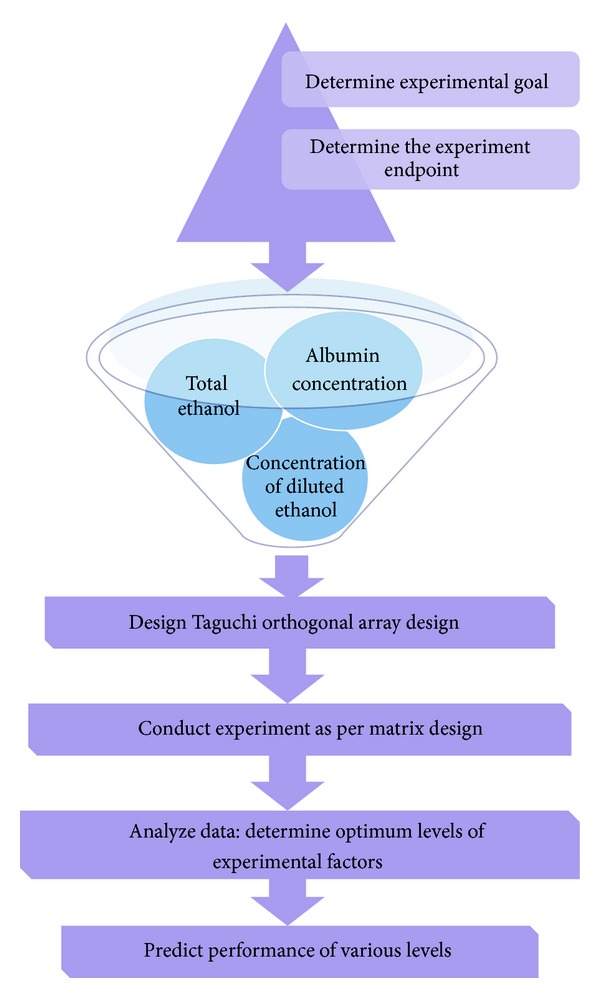
Schematic flow chart showing overview of Taguchi Method.

The Taguchi design has been applied to predict the significant contribution of the design variable(s) and the optimum combination of each variable by conducting experiments on a real-time basis. The modeling that is performed in Taguchi design essentially relates signal-to-noise ratio to the control variables in a “main effect only” approach (Figure 1). This approach enables both multiple response and dynamic problems to be studied by handling noise factors. Taguchi principles and concepts have made extensive contributions to industry by bringing focused awareness to robustness, noise, and quality.

## 2. Materials and Methods

Acetaminophen and gemcitabine HCl was purchased from Sigma Aldrich (USA). BSA and chitosan were purchased from Tokyo Chemical Industries (Tokyo, Japan) and Amresco (OH, USA), respectively. Tripolyphosphate (TPP) was purchased from Strem Chemicals (MA, USA). Dialysis membrane (Spectra/Por 300 KDa MWCO) and membrane filter (Millex pore size 0.45 *μ*m) were purchased from Fisher Scientific UK Ltd. (Loughborough, UK). Corning Transwell polycarbonate membrane inserts (pore size 3.0 *μ*m, membrane diameter 12 mm) and Dulbecco's Modified Eagles Medium (DMEM) were purchased from (Mediatech Inc., VA, USA). Foetal bovine serum (FBS), L-glutamine, nonessential amino acid, 50 IU/mL penicillin and 50 mg/mL streptomycin, and trypsin-EDTA 0.25% were purchased from Gibco BRL, Invitrogen Paisley, UK. All other chemical reagents were analytical grade and obtained from commercial sources. 

### 2.1. Cell Culture

The human lung adenocarcinoma epithelial cell lines, H460 and A549, were grown as monolayer in 75 cm^2^ tissue culture flasks (Greiner Bio-One, Monrew, NC, USA) at 37°C under 5% CO_2_ in RPMI and F12K medium (Life Technologies, Grand Island, NY, USA) supplemented with 10% fetal bovine serum (FBS) and an antibiotic antimycotic solution of penicillin (5000 U/mL), streptomycin (0.1 mg/mL), and neomycin (0.2 mg/mL) (PSN). Cell culture media and PSN stock solutions were purchased from Cellgro (Herndon, VA, USA). Heat inactivated FBS was purchased from Atlanta Biologicals (Lawrenceville, GA, USA). 

### 2.2. Optimization and Selection of Independent Variables: Rationale for Level Selection

BSA nanocarriers were prepared employing the widely reported desolvation technique, with modifications to attain small sized nanocarrier formulation [16, 31]. Three variables, namely, albumin concentration (% w/v), volume of BSA solution to total ethanol ratio (v : v), concentration of diluted ethanol (% v/v), were selected on ground of their paramount influence on particle size distribution of albumin nanocarriers. Briefly, albumin concentration was varied between 1% and 7% w/v taking other parameters constant (volume of BSA solution to total ethanol ratio, 1 : 1 v/v; concentration of diluted ethanol 100% v/v) to decide the approximate range of albumin concentration in formulation that can be engaged for Taguchi orthogonal array design to get reduced particle sized albumin nanocarrier. Similarly, the effect of various levels of volume of BSA solution to total ethanol ratio on nanocarrier formulation was also studied between 1 : 0 and 1 : 1.6 v/v (albumin concentration, 1% w/w; concentration of diluted ethanol, 100% v/v). During our preliminary studies, addition of ethanol as diluted solution was also observed to be influencing particle size. Hence, concentration of ethanol was optimized between 30% and 100% v/v keeping other variables constant (albumin concentration, 1% w/v; volume of BSA solution to total ethanol ratio, 1 : 1 v/v) to attain coacervation of albumin polymer to form albumin nanocarrier.

### 2.3. Taguchi Orthogonal Array Design: Details of Experiments

From the above experiments, it was found that three important factors that influence the size of albumin nanocarrier were albumin concentration, concentration of diluted ethanol, and volume of BSA solution to total ethanol ratio. Hence, in order to minimize the number of experiments, automatic design/analysis Taguchi experiments were employed using MINITAB-16 software (Minitab Inc., PA USA; version XVI). Taguchi orthogonal array table was designed by employing L type orthogonal array (L_9_) by choosing these three parameters that could affect the particle size. The subscript 9 represents Latin square and corresponds to the optimized number of experimental sets to be performed using Taguchi array design to assess the influence of these parameters on each other.  Table 1 shows the parameters and levels used in this experiment. Figure 1 provides a brief overview of the process followed by Taguchi's approach to parameter design [30, 32]. Briefly, 3^3^ Taguchi orthogonal array design (3 parameters and 3 levels) was designed to formulate BSA nanocarrier of small sized range. Briefly, BSA (3%, 4%, and 5% w/v), ethanol dilution (40%, 70%, and 100% v/v), and volume of BSA solution to total ethanol ratio (1 : 0.75, 1 : 0.90, 1 : 1.05 v/v) with size, zeta potential, and entrapment efficiency were considered as dependent variables. For this Taguchi orthogonal array design, APAP was used as model drug to assess the influence of selected parameters on formulation properties. Briefly, APAP (2 mg) was dissolved in 1 mL deionized water (0.22 *μ*m filtered) and was then this solution is injected slowly in the desired solution of BSA (MW: 66478 Da) as per experimental design (Table 1) under continuous stirring at 600 rpm, and the stirring was continued for 45 min. After 45 minutes, ethanol solution was added to each experimental setup as briefed in (Table 1). It should be noted that as against to the reported methodologies [18, 33] that engaged the ethanol addition speed of 1 mL/min, we engaged lower ethanol addition speed of 0.2 mL/min to a less harsh condition for nanocarrier fabrication.

After rigorous optimization of experimental conditions employing APAP as model drug, optimum BSA concentration, ethanol dilution, and volume of BSA solution to total ethanol ratio were found to be 4% w/v, 70% v/v, and 1 : 0.90 v/v, respectively. These conditions were engaged to generate albumin nanocarriers containing potent anticancer drug GEM.

### 2.4. Formulation Development of GEM Loaded Hybrid Nanocarriers

The formulation of hybrid nanocarriers with GEM was achieved by a two-step process as briefed in Figure 4. In step I, the GEM loaded BSA nanocarriers were prepared by modified desolvation technique (M-ANC-G) using the parameters optimized using Taguchi orthogonal array design [16, 27, 34]. Briefly, GEM (2 mg) was dissolved in 1 mL deionized water. The GEM solution was then injected slowly at a speed of 0.2 mL/min in 4% w/v solution of BSA (MW: 66478 Da) under continuous stirring at 600 rpm, and the stirring was continued for 45 min. After stirring continually for 45 min, diluted ethanol (final concentration 70% v/v) was added slowly at a speed of 0.2 mL/min to attain final ethanol concentration of 90% in the mixture. The mixture was stirred for an additional period of 1 hr followed by reduction of stirring speed to 200 rpm to evaporate residual ethanol.

In step II, preformed M-ANC-G was coated with chitosan by solvent gelation method and for this, chitosan solution (0.125% w/v) was added slowly under stirring at 600 rpm for 1 hr. After 1 hr, tripolyphosphate (TPP, 0.125% w/v) was added to crosslink and stabilized the formed M-HNC-G. TPP was engaged as crosslinker in place of well-established glutaraldehyde cross-linker on ground of superior safety and biocompatibility profile of TPP [35]. Finally, the TPP stabilized M-HNC-G were collected by centrifugation (16,000 g, 30 min) employing Vivaspin 500 ultracentrifuge filters with molecular cut-off weight of 300 K Da (Viva Products, Inc., Littleton, MA, USA), and the product was lyophilized using 2% w/v lactose as cryoprotectant [8]. Following conventional methodology, albumin nanocarriers (C-ANC-G) as well as hybrid nanocarriers (C-HNC-G) of GEM were also prepared to assess the influence of these modifications on bio-performance of nanocarriers.

### 2.5. Size, Surface Charge, and Entrapment of GEM Loaded Hybrid Nanocarriers

The particle size and surface charge (zeta potential) of prepared formulations were determined by dynamic light scattering using a NICOMP ZLS 380 analyzer (PSS-NICOMP, Santa Barbara, CA, USA) as described previously by our group [7, 8, 36–40]. The particle size and zeta potential were determined by dispersion of the prepared formulations in PBS at 25°C. All measurements were recorded in triplicate.

### 2.6. Determination of Entrapment Efficiency

Entrapment efficiency was determined by employing Vivaspin 500 ultracentrifuge filters with molecular cut-off weight of 300 K Da (Viva Products, Inc., Littleton, MA, USA). Briefly, GEM loaded formulations (0.5 mL) was placed on top of the Vivaspin filter membrane (molecular weight cutoff 300 K Da) and centrifuged at 13,000 rpm for 15 min. The aqueous filtrate generated at the bottom of Vivaspin 500 ultracentrifuge tubes was subjected to UV spectrophotometric analysis to determine the concentration of unloaded GEM (*λ*_max⁡_  248.6 nm; *ε*_248_ = 0.7053 × 10^4^ L mol^−1^cm^−1^) [13]. The entrapment efficiency (EE) of the GEM within developed formulations was defined as the drug content and was calculated by employing (1) as follows:

(1) $$\begin{array}{c} \%\;\text{Entrapment}\;\text{Efficiency}\;\left(\text{EE}\right)=\frac{X_{1}-X_{2}}{X_{1}}\times 100, \end{array}$$

where *X*_1_ = amount of total GEM taken (mg) and *X*_2_ = amount of free GEM detected after ultracentrifugation (mg).

For APAP assay (*λ*_max⁡  _248.6 nm), similar protocol was followed with slight modifications [41].

### 2.7. *In Vitro* Release of GEM from Various Nanoformulation

*In vitro* release of GEM from the developed formulations was carried out under physiological pH (pH 7.4) using phosphate buffer saline (PBS, pH 7.4) by employing dialysis tube diffusion technique [5, 42–44]. Lyophilized C-ANC-G, M-ANC-G, C-HNC-G, M-HNC-G, and plain GEM were re-suspended in PBS solution and filled inside dialysis membrane bag with molecular weight cutoff of 12 K Da (Sigma, USA). The membrane bags were placed in 50 mL of at PBS pH 7.4 medium. The entire system was maintained at 37 ± 2°C with continuous slow magnetic stirring at 300 rpm. At predetermined time intervals (0.25, 0.5, 1, 2, 4, 6, 8, 12, 24, and 48 hr), 0.5 mL aliquots of dissolution medium were withdrawn and replenished quickly with equivalent volume of fresh medium to maintain the sink condition. The samples were analyzed at each time point using the reported UV spectrophotometric method (*λ*_max⁡_248.6 nm; *ε*_248_ = 0.7053 × 10^4^ L mol^−1^cm^−1^) [13]. The release data were analyzed by plotting time versus percent GEM release to evaluate the release profile of developed formulations.

### 2.8. Stability Studies

Various nanocarriers were also exposed to varying pH conditions (pH 5.4, 6.4, and 7.4) to infer the effect of pH on surface properties of nanocarriers. This stability study was performed with an intention to investigate the influence of pH on size and surface charge of various nanoformulations. For this study, pH 7.4 was selected to mimic the stability of formulation under physiological conditions (blood), whereas pH of 5.4 was selected to mimic the formulation under acidic tumor environment. The inclusion of pH 6.4 was done to view the pH dependent alternation in size and surface charge of nanocarrier [45]. 

### 2.9. Cell-Line Based *In Vitro* Cytotoxicity Assay

The *in vitro* cytotoxicity of free GEM and developed formulations (C-ANC-G, M-ANC-G, C-HNC-G, and M-HNC-G) was evaluated by *MTT *cytotoxicity assay based on measurement of the activity of enzymes present in live cells that reduce *MTT* to give a purple color [42, 43]. This assay was performed on A549 and H460 cell lines. For this, cells (5 × 10^3^) were seeded evenly into 96-well flat-bottomed tissue culture plate (Iwaki; Japan) in DMEM medium (Mediatech Inc., VA, USA) supplemented with 10% FBS (FBS; Sigma, USA) and 1% Penicillin-Streptomycin mixture (Sigma, USA). The cells were incubated for 4 h following in a humidified atmosphere of 5% CO_2_ at 37 ± 0.5°C and formulations were applied as freshly prepared solutions between 0.001 and 100 *μ*M concentrations. After 24 and 48 h, 20 *μ*L *MTT* (5 mg/mL) in PBS (pH 7.4) was added to each well and the plate was incubated for 2 h at 37 ± 0.5°C, allowing viable cells to reduce the *MTT* into purple colored formazan crystals. The formazan crystal was dissolved by adding 100 *μ*L of lysine buffer (TrisHCl, 10 mM; NaCl, 75 mM; EDTA, 10 mM; sodium dodecyl sulphate, 0.5% in water) containing Proteinase K (0.15 mg/mL). The absorbance was measured at 570 nm using ELISA microplate reader (Synergy H1, Bio-Tek, USA) at 37 ± 0.5°C. 

### 2.10. Cell Uptake Assay

Human lung cancer H460 cells (5 × 10^4^) were seeded on six well plates and incubated at 37 ± 0.5°C under 5% CO_2_ for 24 h. The media was removed and incubated with 2 mL of Nile Red loaded formulations (C-ANC-G, M-ANC-G, C-HNC-G, and M-HNC-G) for 1 h. After 1 hr, media was removed, and the resulting cells were washed with PBS and fixed with 2% formaldehyde in the PBS at room temperature for 10 min, followed by washing with PBS twice. The samples were imaged using Axiovert 40 inverted microscope (at magnification of 40x) with Nile Red 554 nm excitation and 635 nm emission wavelength with mercury vapor lamp as light source for fluorescence imaging.

### 2.11. Statistical Analysis

The experiments were conducted in triplicate unless specified, and the data presented as mean ± standard error (SD). Experimental statistics were performed using MINITAB 16 software (Minitab Inc., PA, USA). A One-way analysis of variance (ANOVA) with Tukey's multiple comparison posttest was used in the analysis of differences between the physicochemical properties of nanocarriers with and without chitosan coating (confidence limit of *P* < 0.05). The least significant difference post hoc ANOVA analysis was used in the comparison of particle sizes between different formulations. 

## 3. Result and Discussion

Abnormal angiogenesis is one of the key characteristic features of rapidly proliferating tumor cells, which emerges to meet their raised nutritional and oxygen requirements. These newly formed tumor vasculature are poorly aligned and bear defective endothelial cells with wide fenestrations [23]. It is well reported in the literature that molecules of size <100 nm attain rapid access through these fenestrations availing EPR benefit [24, 46]. Additionally, it was well reported that negatively charged cancer cells interact effectively with positively charged nanoconstructs [47]. The mainstream of the other drug delivery nanocarriers employed for the same function would not readily gain admittance to tumors from the vasculature, owing to their larger diameters (between 100 and 300 nm), which is too large to cross vascular pores [14, 18]. Hybrid nanocarriers formulated by us are of size range *≈*50 nm and hence can get entrance through pores in the vasculature and infuse tumor cells directly [15, 16]. Hence, the major objective of this work was to robustly generate positively charged nanocarrier with size range of <100 nm, so as to attain high intratumoral delivery of anticancer drug by engaging EPR benefit as well as charge-based interactions [48]. Our studies also report systematic design of Taguchi experiment to design BSA and chitosan polymer-based hybrid nanocarriers with accounts on their size, surface charge and entrapment. This study also briefs the influence of negative and positive charged nanocarriers on *in vitro* release, pH-based stability, cell-line-based cytotoxicity, and inverted microscope-based cell uptake study. 

**Figure 2:**
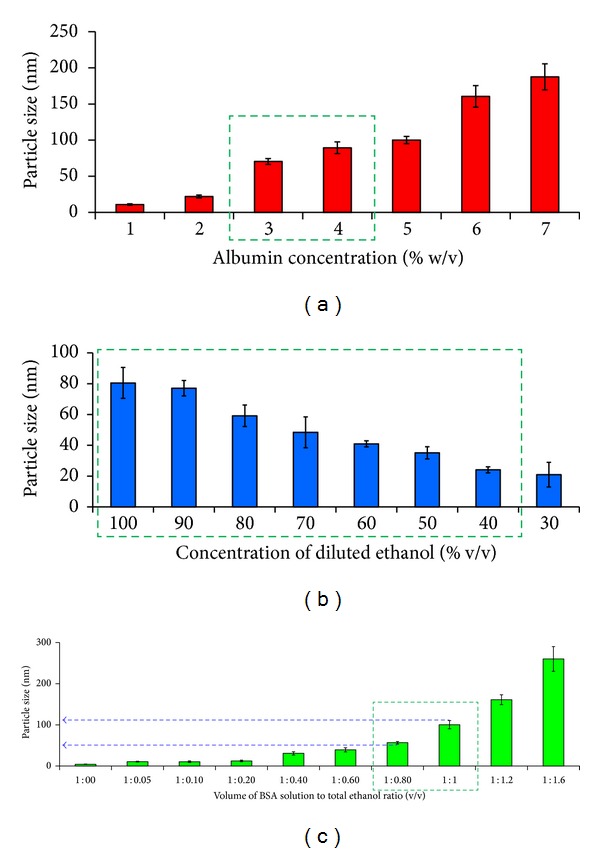
Optimization of (a) BSA concentration (% w/v), concentration of diluted ethanol (% v/v), and (c) volume of BSA solution to total ethanol ratio (v/v). Albumin concentration was optimized between 1% and 7% w/v (at constant volume of BSA solution to total ethanol ratio 1 : 1 v/v; concentration of diluted ethanol 100% v/v), while the effect of volume of BSA solution to total ethanol ratio on nanoparticle formulation was studied between 1 : 00 and 1 : 1.60 v/v (at albumin concentration, 1% w/w; concentration of diluted ethanol, 100% v/v). The concentration of diluted ethanol was optimized between 30% and 100% v/v keeping other variables constant (albumin concentration 1% w/v; volume of BSA solution to total ethanol ratio 1 : 1 v/v). Dotted square box represents the selection window for various levels engaged for Taguchi experiment design. Results are represented as mean ± SD, *n* = 3.

In our preliminary investigations, the BSA concentration and volume of BSA solution to total ethanol ratio used for nanocarrier formulation were shown to influence the particle size of formulation. Therefore, first of all BSA concentration was optimized between 1% and 7% w/v (at volume of BSA solution to total ethanol ratio set at 1 : 1 v/v and concentration of diluted ethanol being set at 100% v/v). The study inferred that BSA concentration range between 3 and 5% w/v to be the best levels and hence we have selected BSA concentration of 3%, 4%, and 5% w/v for Taguchi experimental design (Figure 2). Similarly, the effect of volume of BSA solution to total ethanol ratio for nanocarrier formulation was studied between 1 : 00 and 1 : 1.60 v/v (BSA concentration 1% w/v; concentration of diluted ethanol 100% v/v), where upon 1 : 0.75, 1 : 0.90, and 1 : 1.05 v/v were selected as volume of BSA solution to total ethanol ratio as one of the experimental levels for Taguchi factorial design.

The major amendment made by us in reported methodology for the preparation of albumin nanocarrier includes the inclusion of diluted ethanol to affect coacervation as against pure ethanol used in reported work [31, 49]. This inclusion of diluted ethanol was found to be a major breakthrough in our study, as this factor was found to be significantly affecting the particle size of end formulation. It was anticipated that diluted ethanol will impart less harsh condition for desolvation and nanofabrication thereby allowing mild folding of BSA polymer to generate nanocarriers of <100 nm in contrast to that of reported methodologies that generated nanocarriers of size ranging between 150 and 300 nm [17, 18, 33]. The concentration of diluted ethanol (aqueous) was optimized between 30% and 100% v/v keeping other variables constant (BSA concentration, 1% w/v; volume of BSA solution to total ethanol ratio 1 : 1 v/v). From the outcome of this investigation, 40%, 70%, and 100% v/v concentration of diluted ethanol was selected for Taguchi array design.

**Figure 3:**
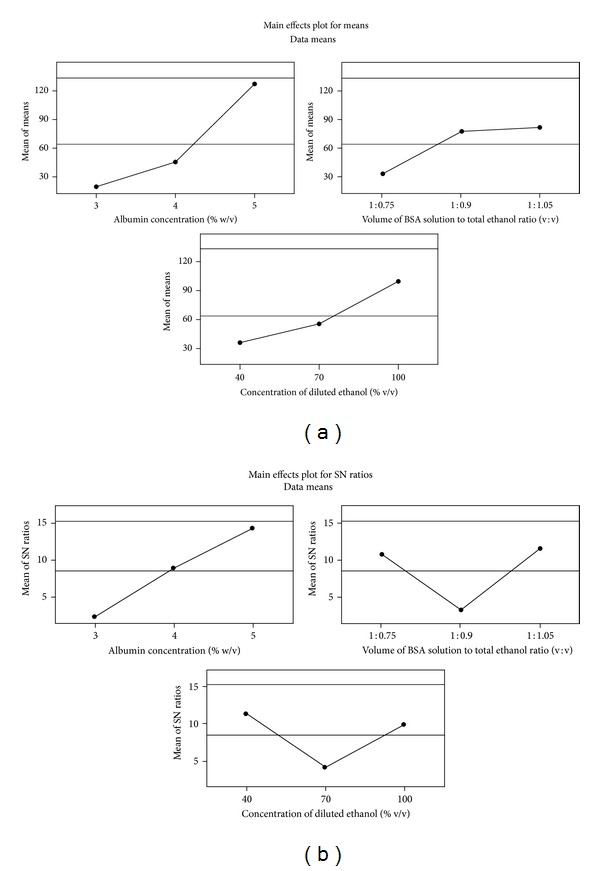
Experimental outcome of Taguchi designed experiments: (a) mean effect plot and (b) mean effect plot for signal-noise (SN) ratio. Signal-to-noise: nominal is best (10*Log10(Ybar**2/s**2)). Optimum formulation conditions: BSA concentration 4% w/w; volume of BSA solution to total ethanol ratio 1 : 0.90 v/v; concentration of diluted ethanol 70% v/v. Ethanol dilution was found to be the most influential factor effecting particle size.

**Figure 4:**
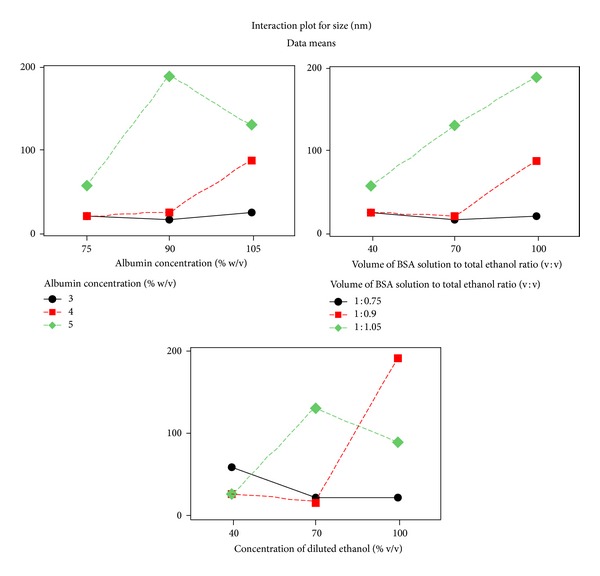
Interaction plots for experimental outcome of Taguchi designed experiments. Optimum formulation conditions: BSA concentration 4% w/w; volume of BSA solution to total ethanol ratio 1 : 0.90 v/v; concentration of diluted ethanol 70% v/v. Ethanol dilution was found to be the most influential factor effecting particle size.

APAP was engaged as model drug for the optimization of these experimental conditions suitable for formulation of small molecules. Following selection of key dependent factors that affect formulation characteristics, a Taguchi orthogonal array design [27, 30, 34] was adopted to identify the optimal conditions as well as to select the parameters having the most principal influence on the particle size of ANC.  Table 1 shows the structure of Taguchi orthogonal array design and the results of measurement are presented in Table 2. The level-average graph of Taguchi designed experiment is illustrated in Figure 3, which shows that data analyzed by the Taguchi method is in good agreement with experimental findings (Table 2). The interaction graph as presented in Figure 4 (figures generated by using MINITAB-16; Minitab Inc., USA) clearly represents the trend of each factor with respect to different levels. The outcome inferred ethanol dilution to be the most influential factor effecting particle size. Although, 3% w/v BSA concentration and 40% v/v ethanolic aqueous solution resulted in small sized nanocarriers of size ranging between 15 and 40 nm, however, these were not selected as optimal parameters owing to production of significantly lower yield of nanocarriers. The optimum formulation conditions that were selected from APAP model drug based Taguchi experimental design were corresponding to the experimental set that engages the BSA concentration 4% w/w; volume of BSA solution to total ethanol ratio 1 : 0.90 v/v, and concentration of diluted ethanol 70% v/v.

**Figure 5:**
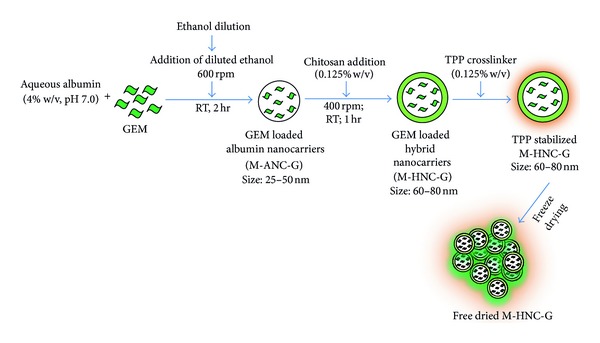
Schematic showing formulation of GEM loaded hybrid nanocarrier (N-HNC-G).

**Figure 6:**
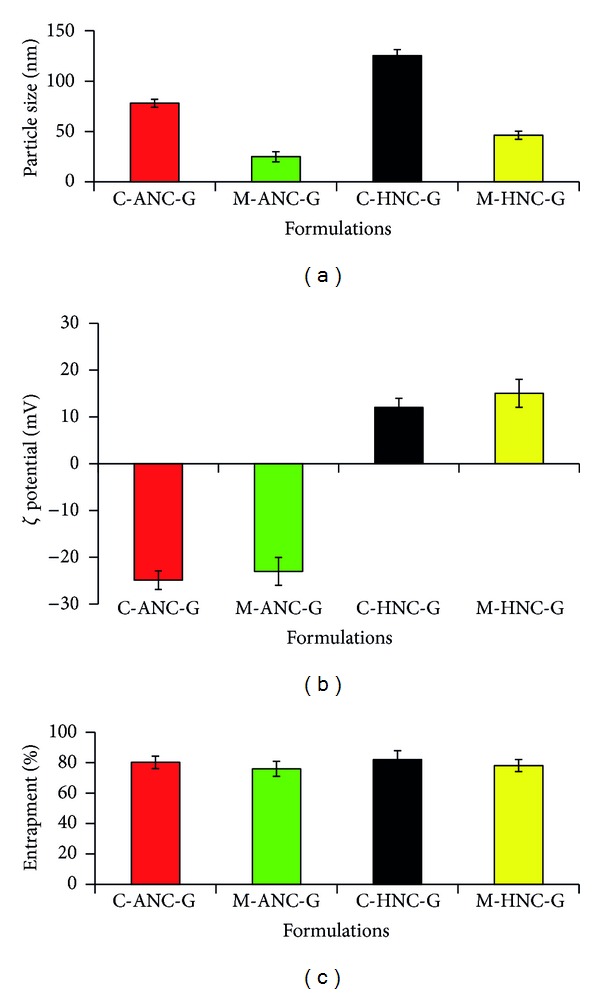
Figure showing (a) particle size, nm; (b) Zeta potential, mV; (c) entrapment % of developed nanocarriers. C-ANC-G and C-HNC-G represents gemcitabine loaded albumin nanocarrier and hybrid nanocarrier, respectively, formulated using conventional method; while M-ANC-G and M-HNC-G represent gemcitabine loaded albumin nanocarrier and Hybrid nanocarrier, respectively, formulated using method modified using Taguchi orthogonal array design method. Results are represented as mean ± SD (*n* = 3).

These optimized experimental conditions were employed to develop GEM loaded ANC (M-ANC-G), which successfully produced M-ANC-G of size 25.07 ± 2.81 nm bearing negative charge (*ζ* = −23.03 ± 1.015 mV) (Figure 5). For comparison, GEM loaded ANC was also prepared using conventional methodology (C-ANC-G; 4% w/v BSA, 1 : 1 v/v volume of BSA solution to total ethanol; undiluted), wherein nanocarriers of 78.01 ± 4.99 nm size (*ζ* = −24.88 ± 1.37 mV) were obtained (Figures 6(a) and 6(b)). Both the ANCs formulated by conventional as well as modified method produced the nanocarriers with similar surface charge (M-ANC-G, *ζ* = −23.03 ± 1.015 mV; C-ANC-G, *ζ* = −24.88 ± 1.37 mV; *P* > 0.05). However, the ANC produced by modified technique has been successful in developing approximately 50% smaller particle as compared to that of ANC produced by conventional methods.

**Figure 7:**
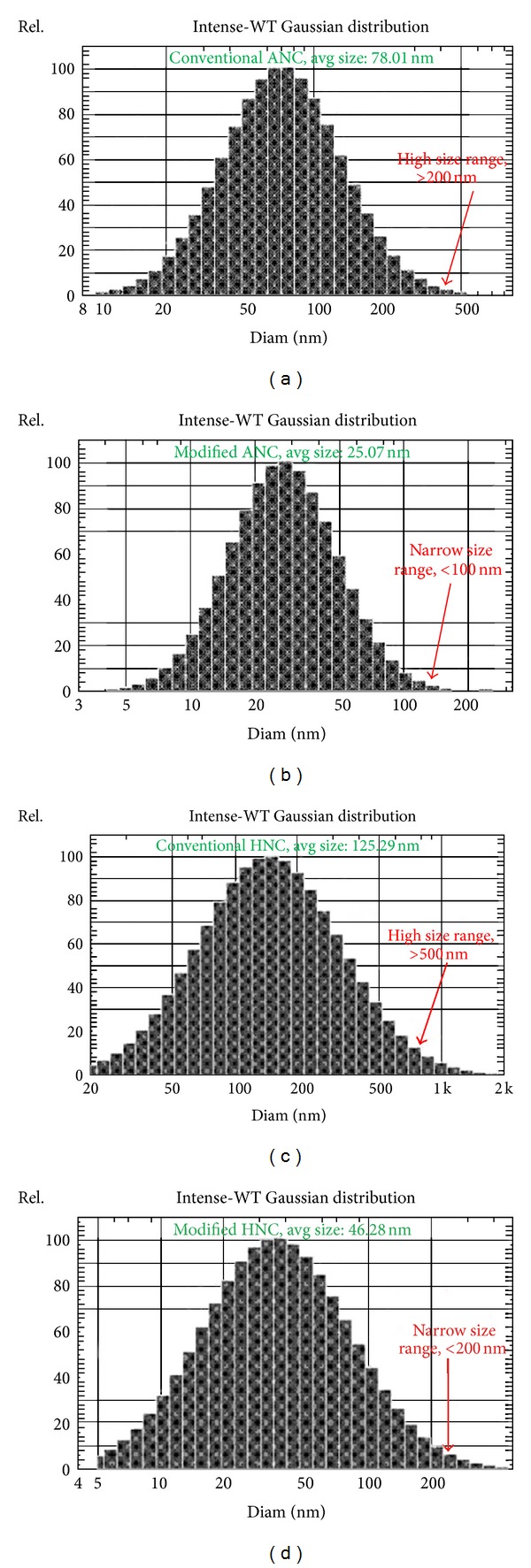
Graph showing particle size distribution of (a) C-ANC-G, (b) M-ANC-G, (c) C-HNC-G, and (d) M-HNC-G. C-ANC-G and C-HNC-G represent gemcitabine loaded albumin nanocarrier and hybrid nanocarrier, respectively, formulated using conventional method, while M-ANC-G and M-HNC-G represent gemcitabine loaded albumin nanocarrier and hybrid nanocarrier, respectively formulated using method modified using Taguchi orthogonal array design method. Particle size and surface charge (zeta potential) were determined in PBS at 25°C by dynamic light scattering using a NICOMP ZLS 380 analyzer (PSS-NICOMP, Santa Barbara, CA, USA) [14]. Results are represented as mean ± SD (*n* = 3).

With an intention to produce positively charged nanocarrier with high cellular binding/internalization as well as to avail aided sustained drug release benefit, hybrid nanocarriers were generated by chitosan layering (solvent gelation method; Figure 5) of modified ANC [31, 49]. For generating comparative data, hybrid nanocarriers were also developed using conventionally produced ANC. The modified formulation method produced formulations with narrow size distribution as compared to that of conventional methodology that generated particles with wide size distribution as evinced from size distribution curve (Figure 7).

The conventional ANC-based hybrid nanocarrier (C-HNC-G) was found to be of 125.29 ± 5.62 nm size (*ζ* = 12.01 ± 0.51), as against small sized hybrid nanocarrier obtained with modified method (M-HNC-G; 46.28 ± 2.21 nm; *ζ* = 15.05 ± 0.39). Upon coating ANC with 0.05% v/v chitosan, its surface charge was observed to be altering drastically from negative charge to positive charge (Figures 6(a) and 6(b)). The shift of charge from negative to positive also confirms the successful layering of chitosan on preformed ANC.

**Figure 8:**
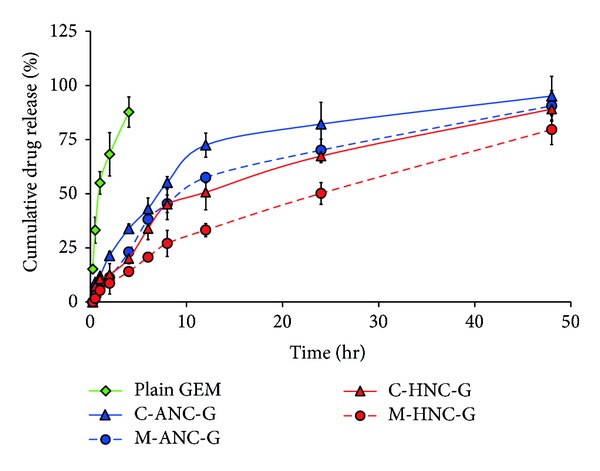
*In vitro* release profile of GEM from various nanocarriers under physiological pH (pH 7.4). Lyophilized formulations were resuspended in PBS solution and filled inside dialysis membrane bag with molecular weight cutoff of 12 K Da (Sigma, USA). The membrane bags were placed in 50 mL of at PBS pH 7.4 medium maintaining the system at 37 ± 2°C with continuous slow magnetic stirring at 300 rpm. At specific time intervals, 0.5 mL aliquots of dissolution medium were withdrawn and analyzed for GEM content. Results are represented as mean ± S.D (*n* = 3).

The entrapment efficacy of C-ANC-G, M-ANC-G, C-HNC-G, and M-HNC-G was found to be 80.2 ± 5.72, 75.95 ± 6.08, 81.94 ± 4.15, and 78.12 ± 3.77%, respectively (Figure 6(c)). The *in vitro* release study performed under physiological pH (PBS, 7.4) inferred slow and sustained and controlled release of GEM. Plain GEM was found to be releasing almost completely within 4-5 hr; on the other hand, it took more than 24 hr to release almost of *≈*75% GEM from ANC. The modified method that generated M-ANC-G was found to be more efficient in drug retention (sustained effect) as compared to that of ANC generated by using conventional method (C-ANC-G). After 2 hr, M-ANC-G released 11.28 ± 2.05% GEM as against 21.40 ± 1.95% GEM released by C-ANC-G (Figure 8). This outcome is attributed to the efficient packing as well as loading of GEM inside ANC in M-ANC-G due to existence of less harsh loading condition provided by using diluted ethanol. At 0.5 and 1 hr time points, C-ANC-G released 9.15 ± 0.28% and 12.01 ± 1.11% GEM, as against 2.81 ± 0.15% and 6.06 ± 0.19%, respectively, by M-ANC-G. This infers high burst release effect in case of conventional method formulated ANC as compared to our novel modified method. 

Furthermore, it was observed that hybrid nanocarriers produced by chitosan layering of ANC further slowed down the release of GEM (Figure 8). However, in this case also release was observed to be more sustained and slow in case of M-HNC-G as compared to C-HNC-G. At 12, 24, and 48 hr time points, release of GEM from M-HNC-G was found to be 33.24 ± 3.87%, 50.15 ± 5.12%, and  79.62 ± 7.08%, respectively, as against to 50.65 ± 8.07%, 67.39 ± 3.15%, and 89.06 ± 5.93%, respectively, with C-HNC-G. This effect is attributed to the efficient GEM retention capability of M-ANC-G, which was engaged form M-HNC-G. Also, this effect can be attributed to efficient chitosan coating due to availability of high surface area with M-ANC-G due to its significantly (*P* < 0.001) small size of 25.07 ± 2.81 nm (*ζ* = −23.03 ± 1.015 mV). 

**Figure 9:**
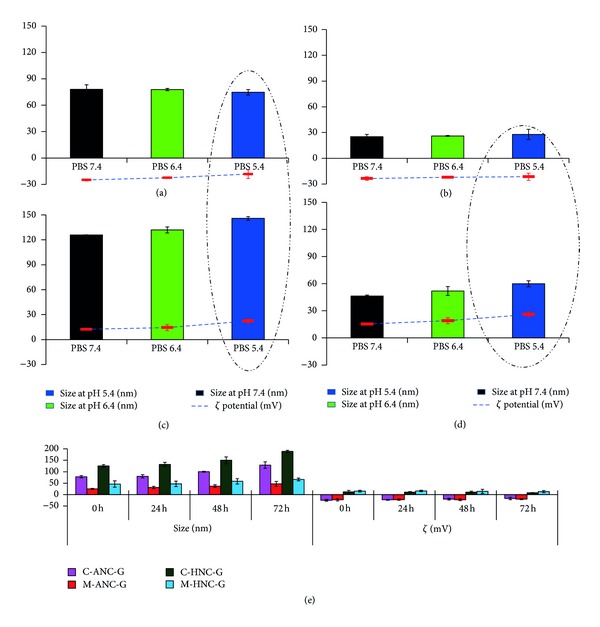
Figure showing stability of *various *formulations: (a) C-ANC-G, (b) M-ANC-G, (c) C-HNC-G, and (d) M-HNC-G with respect to size (nm) and zeta potential (mV) under influence of varying pH between 7.4 and 5.4. C-ANC-G and C-HNC-G represent gemcitabine loaded albumin nanocarrier and hybrid nanocarrier, respectively, formulated using conventional method, while M-ANC-G and M-HNC-G represent gemcitabine loaded albumin nanocarrier and hybrid nanocarrier, respectively, formulated using method modified using Taguchi orthogonal array design method. Dashed circle highlights significant changes in size of nanocarriers at pH 5.4. Results are represented as mean ± SD (*n* = 3).

The pH dependent alternation in particle size was assessed to determine the change in biopharmaceutical properties of these nanoformulations under physiological conditions (pH 7.4) as well as in acidic tumorous environment (pH 5.4) [45]. On moving from physiological pH to acidic environment, an insignificant (*P* > 0.005) change in particles size was observed in cases of C-ANC-G and M-ANC-G; however, slight rise in zeta potential was noticed. In case of C-ANC-G on moving from pH 7.4 to 5.4, its surface charge altered from −24.88 ± 1.08 mV to −18.27 ± 0.97 mV, while in case of M-ANC-G, it reduced from −23.6 ± 0.15 mV to −21.5 ± 1.74 mV (Figures 9(a) and 9(b)).

On the other hand, 15.81 ± 0.49% and 29.23 ± 1.01% increment in size of C-HNC-G and M-HNC-G formulation was noticed when the pH of incubation milieu was altered from 7.4 to 5.4. In similar fashion, effect of pH change on net surface charge was also assessed, wherein a significant enhancement (*P* < 0.05) in zeta potential was observed with decrement in pH. Both the hybrid nanocarriers showed more than 70 ± 2.19% increment in surface charge with decrease of pH from 7.4 to 5.4 (Figures 9(c) and 9(d)), which can be ascribed to the protonation of chitosan under conditions of low pH (pH 5.4) [50]. This protonation of amine functionalities present on chitosan is possibly the key reason behind increase in particle size of the formulation. On storage, the optimized M-ANC-G and M-HNC-G were found to be comparatively more stable (*P* < 0.05) as compared to those of C-ANC-G and C-HNC-G, respectively, in terms of their retention of particle size and surface charge (Figure 9(e)).

The cytotoxicity studies were performed on A549 and H460 cell lines employing *MTT* cytotoxicity assay performed at 24 and 48 hr time points after the formulation treatment. In all the cases, the treatment of placebo ANC as well as HNC showed >95% viability of cells clearly demonstrating the safe and non-toxic nature of and safety building blocks of the nanocarriers (BSA, chitosan, and TPP). It is envisages that the use of safe, biocompatible, and biodegradable albumin and chitosan-based systems will help in the translation of developed nanocarriers for further human administration.

**Figure 10:**
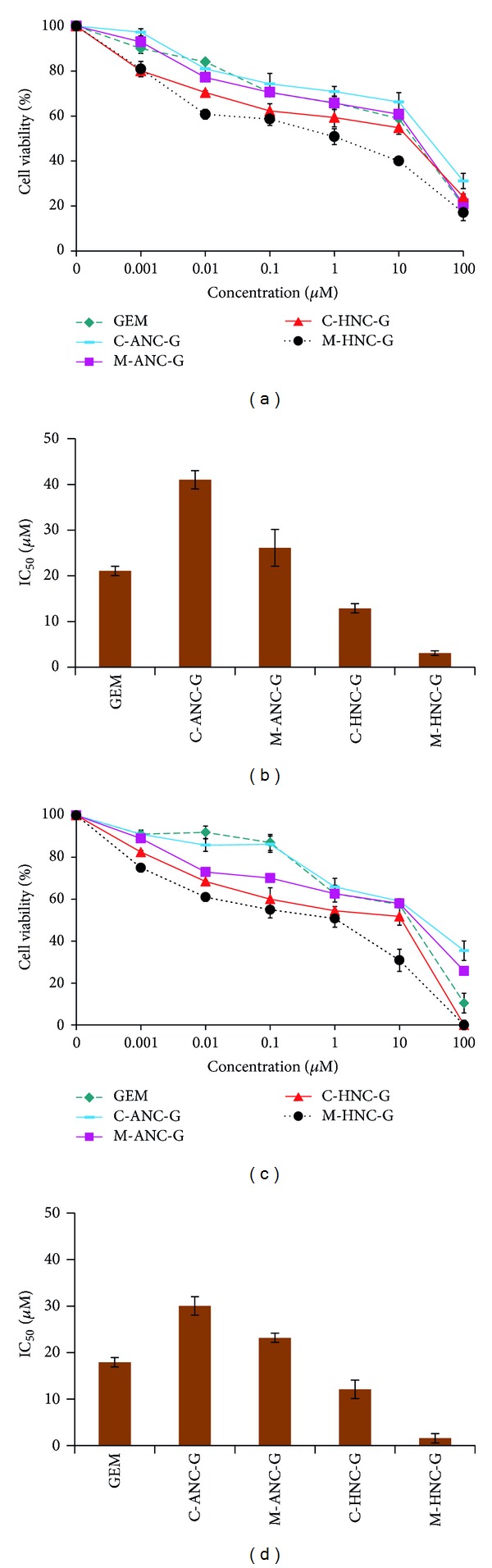
Showing %cell viability and IC_50_ values observed after ((a)-(b)) 24 hr and ((c)-(d)) 48 hr, following treatment of various formulations in A549 lung cancer cell line. Cell viability was performed on 5 × 10^3^ A549 (lung cancer cell lines) cells in F12K medium supplemented with 10% FBS and 1% Penicillin-Streptomycin mixture. Cell incubation was done in a humidified atmosphere of 5% CO_2_ at 37 ± 0.5°C. All formulations were applied as freshly prepared solutions between 0.001 and 100 *μ*M concentrations. The absorbance of formazan crystals dissolved in lysis buffer was measured at 570 nm using ELISA microplate reader at 37 ± 0.5°C. Results are represented as mean ± S.D (*n* = 3).

In A549 cells, 24 hr cytotoxicity assays revealed the IC_50_ of GEM to be 21.07 ± 1.15 *μ*M. Notably, the delivery of GEM using albumin-based GEM formulations showed higher IC_50_ value as compared to GEM solution. C-ANC-G and M-ANC-G showed IC_50_ value of 40.11 ± 2.13 *μ*M and 27.05 ± 4.92 *μ*M, respectively, which is higher than IC_50_ value of naked GEM (21.07 ± 1.15 *μ*M). This can be ascribed to the negative charge associated with basic BSA nanocarriers (C-ANC-G, *ζ* = −24.88 ± 1.37 mV; M-ANC-G, *ζ* = −23.03 ± 1.015 mV) that might be interfering with interaction as well as uptake of nanocarrier inside negatively charged cells [19, 51] Under similar experimental conditions, GEM loaded hybrid nanocarriers were also tested for their cytotoxicity potentials (Figure 10), whereupon significantly low IC_50_ of 12.89 ± 1.14 *μ*M and 3.08 ± 0.56 *μ*M was observed for C-HNC-G and M-HNC-G, respectively. This corresponds to 38.82 ± 1.27% and 92.48 ± 3.78% reduction in IC_50_ value of GEM following its treatment via C-HNC-G and M-HNC-G, respectively. This clearly demonstrates the superiority of formed hybrid nanocarriers generated by modified desolation methodology. Similar observations were made at 48 hr cytotoxicity assay and brief overview of change in IC_50_ of GEM with various formulations (Figure 10). After 48 hr, significant reduction in IC_50_ values of C-HNC-G and M-HNC-G was observed (in IC_50_ values of 12.08 ± 0.24 *μ*M and 1.57 ± 0.03 *μ*M, respectively.

**Figure 11:**
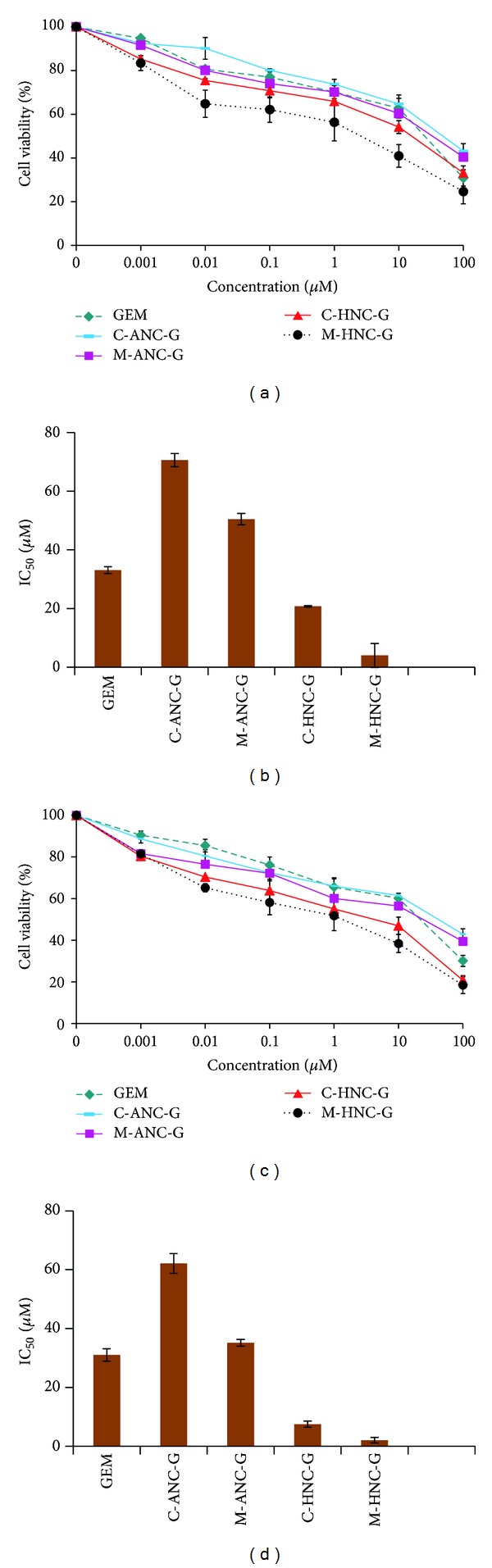
Showing %cell viability and IC_50_ values observed after ((a)-(b)) 24 hr and ((c)-(d)) 48 hr, following treatment of various formulations in H460 lung cancer cell line. Cell viability was performed on 5 × 10^3^ H460 (Lung cancer cell lines) cells in RPMI medium supplemented with 10% FBS and 1% Penicillin-Streptomycin mixture. Cell incubation was done in a humidified atmosphere of 5% CO_2_ at 37 ± 0.5°C. All formulations were applied as freshly prepared solutions between 0.001 and 100 *μ*M concentrations. The absorbance of formazan crystals dissolved in lysis buffer was measured at 570 nm using ELISA microplate reader at 37 ± 0.5°C. Results are represented as mean ± S.D (*n* = 3).

**Figure 12:**
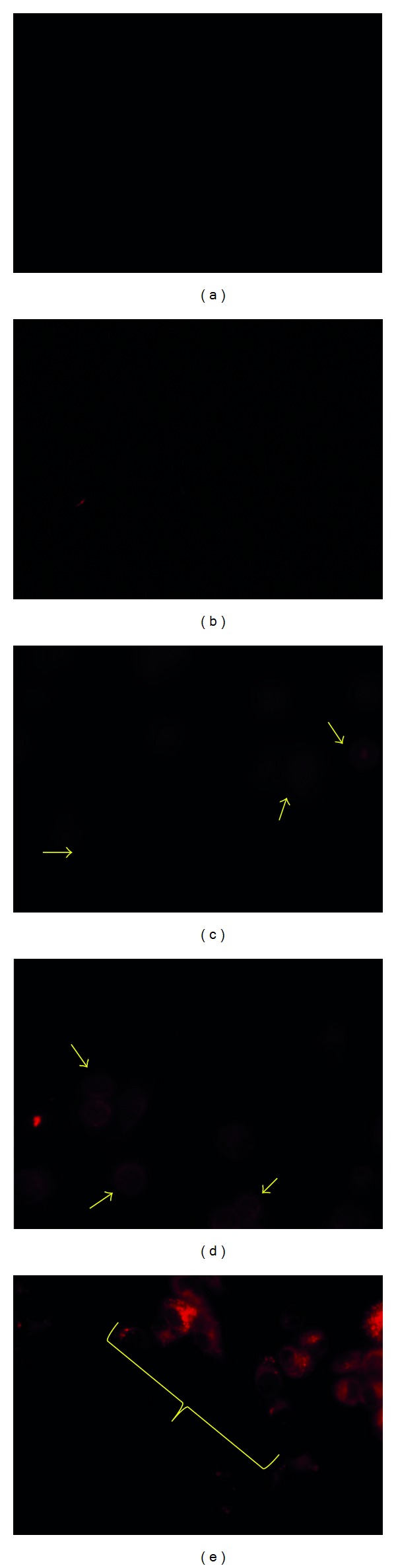
Images depicts the cell uptake of Nile Red loaded: (a) control, (b) C-ANC-G, (c) M-ANC-G, (d) C-HNC-G, and (e) M-HNC-G formulation treated cells taken after 2 hr (at 40x magnification). For this assay Lung Cancer H460 cells (5 × 10^4^) were seeded on six well plates and incubated at 37 ± 0.5°C under 5% CO_2_ for 24 h. The cells were treated with various Nile Red loaded formulations for 1 hr. After 1 hr media was removed and the resulting cells were washed with PBS and fixed with 2% formaldehyde in the PBS at room temperature for 10 min, followed by washing with PBS twice. The samples were imaged using Axiovert 40 inverted microscope (magnification 40x) with Nile Red 554 nm excitation and 635 nm emission wavelength with mercury vapor lamp as light source for fluorescence imaging. Arrow head indicates cells with marked fluorescence.

In H460 cell lines, 24 hr cytotoxicity assay inferred IC_50_ value of GEM, C-ANC-G, and M-ANC-G to be 33.08 ± 1.15, 70.54 ± 2.25, and 50.46 ± 1.899 *μ*M, respectively. The IC_50_ value of GEM was found to be significantly reduced with M-HNC-G (4.01 ± 0.07 *μ*M) by a factor of 87.05 ± 3.17% as compared to GEM solution (Figure 11). To investigate the cellular uptake potential of various nanoformulations under investigation, cell uptake assay was also performed by employing NileRed loaded nanoformulations. In the same line of cytotoxicity assay, cell uptake assay inferred higher uptake of M-HNC-G as compared to other nanoformulations (C-ANC-G, M-ANC-G, and C-HNC-G) under investigation (Figure 12). Results of our investigation robustly support hybrid nanocarrier approach to be efficient in delivering drugs to cancer cell [52].

It is anticipated that this technique can be extended to deliver bioactives of vivid origin ranging from drugs, proteins, peptides, to siRNA and genes with appropriate modifications in proposed methodology. In the midst, when other drug delivery nanocarriers would not readily gain admittance to tumors from the vasculature owing to higher particle size (between 50 and 300 nm) [47, 48], hybrid nanocarriers formulated by us (M-HNC-G; 46.28 ± 2.21 nm; *ζ* = 15.05 ± 0.39) would get entrance through pores in the vasculature and infuse tumor cells directly [53]. In addition to particle size, positive surface charge of HNC will further aid in cellular binding as well as intracellular internalization inside negatively charged cancer cells.

## 4. Conclusion

The core objective of this work was to develop and investigate an effective drug delivery strategy to effectively deliver anticancer drugs with compliance of all issues associated to it and improve the outcome of treatment. Albumin- and chitosan-based innovative hybrid nanocarriers were formulated and reported in this work. Formulation parameters for hybrid nanocarriers were optimized employing APAP as model drug; following these optimized conditions, GEM loaded hybrid nanocarriers were formulated and characterized in terms of particle size, zeta potential determination, *in vitro* release, stability assay, cell viability assay, cell uptake assay and biomedical tests to establish the suitability of this approach in drug delivery and therapy. The results indicate that HNC approach significantly enhances the anticancer potential of loaded GEM. It is encouraging that our data suggest albumin and chitosan biodegradable and biocompatible polymer-based HNC to be efficient in inhibiting cancer cells growth as compared to GEM solution as well as negatively charged nanocarriers (M-ANC-G). Most importantly, treatment of HNC with H460 cells showed remarkable improvement in cell uptake and deposition as compared to that of unmodified as well as conventionally produced nanocarriers. It is envisioned that these HNC's can be utilized to selectively deliver the loaded therapeutics to result in a high drug concentration at desired site of action. Furthermore, it is envisaged that other bioactive compounds (such as antibiotics, anticancer drugs, plasmids, gene, etc.) can be loaded into HNC employing the same formulation strategy as reported in this paper. These results with HNC are promising, but further *in vivo* investigations to explore the efficacy and safety of nanocarriers are necessary to optimize their use in various pharmaceutical applications.

## Figures and Tables

**Table 1:** Factors, variables, and their levels employed in Taguchi orthogonal array design.

Factors	Levels		
	1	2	3
Albumin concentration (% w/v)	3	4	5
Concentration of diluted ethanol (% v/v)	40	70	100
Volume of BSA solution to total ethanol ratio (v/v)	1 : 0.75	1 : 0.90	1 : 1.05

**Table 2:** Experimental data values for particle size and entrapment of albumin nanoparticles obtained by Taguchi orthogonal array table of L9 Design.

Albumin Concentration (% w/v)	Volume of BSA solution to total ethanol ratio (v/v)	Concentration diluted ethanol (% v/v)	Size (nm)	Entrapment efficiency (%)
3	1 : 0.75	100	20.01 ± 1.17	61.06 ± 1.94
3	1 : 0.90	70	15.72 ± 0.83	76.87 ± 2.03
3	1 : 1.05	40	24.69 ± 0.69	69.95 ± 3.84
4	1 : 0.75	70	20.14 ± 0.86	72.38 ± 2.42
4	1 : 0.90	40	25.65 ± 1.71	70.98 ± 1.06
4	1 : 1.05	100	88.90 ± 3.08	78.64 ± 1.97
5	1 : 0.75	40	58.17 ± 1.95	61.75 ± 0.84
5	1 : 0.90	100	191.53 ± 14.96	86.97 ± 2.71
5	1 : 1.05	70	130.69 ± 5.27	83.01 ± 2.09

Particle size and surface charge (zeta potential) were determined in PBS at 25°C by dynamic light scattering using a NICOMP ZLS 380 analyzer (PSS-NICOMP, Santa Barbara, CA, USA) [8]. Entrapment efficiency was determined by employing Vivaspin 500 ultracentrifuge filters with molecular cut-off weight of 300 K Da (Viva Products, Inc., Littleton, MA, USA). Aqueous filtrate generated at the bottom of Vivaspin 500 ultracentrifuge tubes was subjected to UV spectrophotometric analysis to determine the concentration of GEM (*λ*_max⁡_ 248.6 nm; *ε*_248_ = 0.7053 × 10^4^ L mol^−1^ cm^−1^) [13].

Results are presented as mean ± SD, *n* = 3.
